# IFN‐λ3 polymorphism indirectly influences NK cell phenotype and function during acute HCV infection

**DOI:** 10.1002/iid3.122

**Published:** 2016-08-16

**Authors:** Marion Depla, Sandy Pelletier, Nathalie Bédard, Camille Brunaud, Julie Bruneau, Naglaa H. Shoukry

**Affiliations:** ^1^ Centre de Recherche du Centre Hospitalier de l'Université de Montréal (CRCHUM)MontréalQuébecCanada; ^2^ Département de microbiologie et immunologieFaculté de médecineUniversité de MontréalMontréalQuébecCanada; ^3^ Département de médecine familiale et de médecine d'urgenceFaculté de médecineUniversité de MontréalMontréalQuébecCanada; ^4^ Département de médecineFaculté de médecineUniversité de MontréalMontréalQuébecCanada

**Keywords:** Hepatitis C, NK cells, Type III IFN

## Abstract

**Introduction:**

Polymorphisms in the type III interferon IFN‐λ3 and the killer cell immunoglobulin‐like receptor (KIR) genes controlling the activity of natural killer (NK) cells can predict spontaneous resolution of acute hepatitis C virus (HCV) infection. We hypothesized that IFN‐λ3 polymorphism may modulate NK cell function during acute HCV.

**Methods:**

We monitored the plasma levels of type III IFNs in relation to the phenotype and the function of NK cells in a cohort of people who inject drugs (PWID) during acute HCV infection with different outcomes.

**Results:**

Early acute HCV was associated with high variability in type III IFNs plasma levels and the favorable IFN‐λ3 CC genotype was associated with higher viral loads. Reduced expression of Natural Killer Group Protein 2A (NKG2A) was associated with lower IFN‐λ3 plasma levels and the CC genotype. IFN‐γ production by NK cells was higher in individuals with the CC genotype during acute infection but this did not prevent viral persistence. IFN‐λ3 plasma levels did not correlate with function of NK cells and IFN‐λ3 prestimulation did not affect NK cell activation and function.

**Conclusions:**

These results suggest that IFN‐λ3 polymorphism indirectly influences NK cell phenotype and function during acute HCV but other factors may act in concert to determine the outcome of the infection.

## Introduction

The majority of individuals acutely infected with hepatitis C virus (HCV) develop chronic infection. Acute infection outcome is determined by the interplay between the virus, the virus‐specific immune response, and the host genetics such as polymorphisms in the IL28B/IFN‐λ3 locus (encoding for the type III interferon IFN‐λ3), the killer cell immunoglobulin‐like receptor (KIR) and HLA‐C genes controlling the function of natural killer (NK) cells (reviewed in 1, 2). Types I and III IFN responses are induced early during acute HCV. IFN‐λ3 single nucleotide polymorphism (SNP) (rs12979860) was associated with spontaneous HCV clearance 3, 4 with the favorable genotype designated as CC and the non‐favorable as TT. This SNP is located upstream of the IL28B (IFN‐λ3), IL28A (IFN‐λ2), and IL29 (IFN‐λ1) genes and it was postulated that it may control the transcription or the expression levels of type III IFNs but no clear association was established (reviewed in 5). Type I IFNs were minimally detected in the liver and plasma of chimpanzees during acute HCV infection 6, 7. In contrast, type III IFNs were strongly induced and their expression levels correlated with ISG expression in the liver and viremia in acutely infected chimpanzees 6. In humans, IFN‐λ1 was higher than IFN‐λ2/3 during acute HCV and correlated with spontaneous resolution of HCV 8. IFN‐λ3 rs12979860 SNP is in linkage disequilibrium of the coding sequence of another SNP upstream of IL28B controlling the expression of a relatively new member of the type III IFN family, IFN‐λ4, where reduced IFN‐λ4 activity was associated with improved HCV clearance and reduced expression of ISGs 9, 10, 11.

NK cells are one of the earliest innate immune effectors against viral infections and are enriched in the liver (reviewed in 12). Two NK cell subsets can be distinguished based on their differential expression of CD56 and CD16: immunoregulatory CD3^−^CD56^bright^CD16^−^ and cytotoxic CD3^−^CD56^dim^CD16^+^. Activity of NK cells is controlled by a combination of activating and inhibitory receptors including KIRs, lectin‐like (NKG2A‐E), and natural cytotoxicity receptors (NKp30, NKp44, and NKp46) (reviewed in 13). NK cells are activated and may contribute to protection in HCV‐exposed uninfected individuals 14, 15. NK cells were activated in peripheral blood during acute HCV infection irrespective of infection outcome 16, 17, 18 and NK cell activity correlated with the magnitude of adaptive T cell responses suggesting that cross‐talk between the two arms of the immune system is an important determinant of the infectious outcome 17. Polymorphism in both IFN‐λ3 and KIRs synergize to predict the outcome of acute infection or response to IFN‐based therapies 19, 20 and altered NK cell effector functions in chronic hepatitis C were associated with IFN‐λ3 polymorphism 21 suggesting an active role for IFN‐λ3 in NK cell activation. IFN‐λ3 was also recently reported to influence response to an influenza vaccine 22, underscoring the influence of IFN‐λ3 on innate and/or adaptive immune responses. Although the level of expression of its specific Interleukin 28 Receptor Alpha (IL28Rα) on lymphocytes and specifically NK cells is controversial 19, 23, 24, 25, 26, 27, 28, its absence compromises NK cell function in vivo 29.

The exact role of IFN‐λ3 during acute HCV and the mechanism by which it acts to influence infection outcome remain undefined. We hypothesized that polymorphisms in IFN‐λ3 may directly and/or indirectly regulate the activation and function of NK cells during acute HCV and their capacity to control HCV infection. We demonstrate that type III IFNs plasma levels were variable during early acute HCV irrespective of the IFN‐λ3 genotype. Nevertheless, CC IFN‐λ3 genotype was predictive of higher viral load during early acute infection and indirectly influenced NKG2A expression and IFN‐γ production by NK cells.

## Materials and Methods

### Study subjects

Subjects with acute HCV infection (*n* = 28), HCV‐negative “Naïve” (*n* = 20) and HCV‐exposed uninfected subjects (*n* = 9) were recruited among high‐risk people who inject drugs (PWID) participating in the Montreal Acute Hepatitis C Cohort study (HEPCO) 30, 31. Clinical characteristics and demographics are listed in Table 1. All subjects provided informed consent. The study was approved by the institutional ethics committee (Protocol SL05.014) and conducted according to the ethical guidelines of the 1975 Declaration of Helsinki. Acute HCV infection and estimated time of infection were defined as previously described 17. Spontaneous viral resolution (*n* = 9) or chronic infection (*n* = 19) was defined as the absence or presence of HCV RNA, respectively, at 6 months post estimated date of HCV infection. Exposed uninfected (*n* = 9) are PWID who have admitted sharing a needle or injection materials with an HCV‐infected individual but remained HCV RNA and HCV antibody negative. In this study, four time points representing the four phases of HCV infection were analyzed subject to the availability of samples from each patient: i) baseline, defined as time before HCV infection or reported needle sharing for exposed uninfected; ii) early acute, defined as 2 ± 1 month after the estimated time of infection and 1.5 month (range: 1–3 months) after needle sharing for exposed uninfected; iii) late acute phase, defined as 6 ± 2 months; and iv) Long‐term follow‐up, defined as 17 ± 6 months after the estimated time of infection. All patients tested negative for human immunodeficiency virus (HIV) and hepatitis B virus (HBV) and were either medically ineligible for or declined antiviral therapy. Qualitative HCV RNA tests were performed using an automated COBAS Ampli‐Prep/COBAS Amplicor HCV test, version 2.0 (sensitivity <50 IU/ml) (Roche Molecular Systems, Inc., Branchburg, NJ). Additional HCV RNA quantification was performed using an in‐house quantitative real‐time reverse transcription‐PCR assay, as previously described 32.

**Table 1:** Demographics and clinical characteristics of study subjects

	Chronic (C) *n* = 19	Resolver (R) *n* = 9	Naive PWID *n* = 20	Exposed uninfected (EU) *n* = 9	Healthy donors (HD) *n* = 20
Sex (M/F)	17/2	7/2	16/4	7/2	8/12
Age (years) (median)	29	26	36	27	31
HCV genotypes (1/1a/2b/3a/ND)	2/5/‐/11/1	2/1/1/3/2	NA	NA	NA
IFN‐λ3 rs12979860 (CC/*T)	10/9	5/4	10/10	4/5	11/9
IFN‐λ4 ss469415590 (TTTT/*dG/ND)	14/3/2	6/3	13/7	4/5	8/2/10
HCV RNA (IU/ml) (median (IQR))^a^	5.22 × 10^5^ (5.75 × 10^4^–5.39 × 10^6^)	7.02 × 10^5^; (6.16 × 10^5^–9.31 × 10^6^)	NA	NA	NA
ALT level (U/L) (median; IQR)	121; 74–382	164; 37–1650	ND	ND	ND

NA, not applicable; ND, not done; IQR, interquartile range.

^a^ Individuals with detectable viral load (*n* = 15).

### IFN‐λ3 and IFN‐λ4 SNPs genotyping

IFN‐λ3 genotyping was performed as previously described 33. Participants were stratified into CC for those homozygous for the favorable C allele or *T for those heterozygous or homozygous for the unfavorable T allele. IFN‐λ4 genotyping was adapted from Prokunina‐Olsson et al. 9. Isolated genomic DNA was PCR amplified with Phusion Taq polymerase, Buffer 5X GC (Thermo Fisher Scientific, Ottawa, ON, Canada) using Reverse Primer 5′ GCCTGCTGCAGAAGCAGAGAT 3′ 9 and Forward Primer 5′ GAACGGGTGTATGGGAACC 3′ 34 in a total volume of 20 µl per reaction. PCR conditions were as follows: Initial denaturation cycle at 98°C for 30 sec, 30 amplification cycles of 98°C for 10 sec, 62°C for 10 sec, and 72°C for 1 min. A final extension step at 72°C for 7 min was applied. The purified PCR amplified fragments were sequenced by the Sanger Sequencing Service using Applied Biosystem's 3730xl DNA Analyzer technology at the McGill University and Génome Québec Innovation Centre, Montréal, QC, Canada. The sequencing chromatogram was read to obtain the genotype and to discriminate between homozygotes and heterozygotes.

### IFN‐λ3 and IFN‐λ1 ELISA

Commercial ELISA kits were used to quantify IFN‐λ3 (R&D Systems, Minneapolis, MN) and IFN‐λ1 (eBioscience, San Diego, CA) in plasma samples collected in EDTA, according to the manufacturer's protocols. The lower detection limits of the kits were 50 and 30 pg/ml, respectively.

### Multiparametric phenotypic characterization of NK cells by flowcytometry

All flow cytometry assays were performed on cryopreserved peripheral blood mononuclear cells (PBMCs). Multiparametric phenotypic characterization of NK cells was performed as previously described 17. Anti‐IL28Rα‐phycoerythryin(PE) (R&D Clone 601106) was also used.

### NK cells functional assays

PBMCs (1.5–2 × 10^6^) were incubated with anti‐CD107a antibody and either culture medium as a negative control or the MHC class‐I negative K562 leukemia target cell line at a ratio of 5:1 at 37°C in R‐10 medium (RPMI medium supplemented with 10% FBS). Following 1 h of stimulation, 10 ug/ml of brefeldin A (Sigma–Aldrich, St‐Louis, MO) and 6 μg/ml of monensin sodium salt (Sigma–Aldrich) were added, and cells were then incubated for a total of 6 h. Cells were washed with FACS buffer, stained for viability and cell surface antigens, then permeabilized using BD Cytofix/Cytoperm solution (BD Bioscience, San Jose, CA). Cells were then stained with anti‐IFN‐γ antibody for 30 min, washed twice in BD Perm/Wash buffer (BD Biosciences), and fixed in FACS fix buffer. For analysis, cells were gated on viable CD3^−^CD56^bright^CD16^−^, or CD3^−^CD56^dim^CD16^+^ NK cells (Fig. 2A). Percent‐specific expression is calculated as the background‐adjusted function in the presence or absence of the target cell line K562.

### Stimulation of PBMCs and purified NK cells with IFN‐α and IFN‐λ3

PBMCs were thawed and rested for 24 h at 2 × 10^6^ cells/ml in R‐10 or the NK cell population was isolated by negative selection using MACS NK cell isolation kit (Miltenyi Biotec, Auburn, CA) and also rested for 24 h. Purity of the NK cell fraction was assessed by flow cytometry. NK cells were resuspended at 2 × 10^6^ cells/ml in RPMI media supplemented with 5% human serum (Sigma H4522), 100 U/ml penicillin, 100 μg/ml streptomycin, glutamine (2 mM) (Wisent, St‐Bruno, QC, Canada), non‐essential amino acids 1× (Wisent), sodium pyruvate (1 mM) (Wisent), and 2‐mercaptoethanol (5 × 10^−5^ M) (Sigma) at 2 × 10^6^ cells/ml. PBMCs (1.5–2 × 10^6^ for functional assay, 1 × 10^6^ for phenotypic assay) and NK cells (0.5–1.25 × 10^5^) were stimulated overnight in corresponding medium with or without 100 ng/ml of IFN‐λ3 (R&D) or 1000 IU/ml of IFN‐α (PBL Interferon Source). PBMCs or NK cells for the functional assay were then incubated with anti‐CD107a antibody and either culture medium as a negative control, or K562 at a ratio of 5:1 (PBMCs) or 1:1 (NK) at 37°C. Phenotypic characterization of NK cells and intracellular cytokine staining were then performed as described above. We also performed phenotypic characterization of dendritic cells (DC) (CD3^−^CD14^−^CD16^−^HLADR^+^CD11c^+^) and monocytes (CD3^−^CD14^+^CD16^+^) populations using Anti‐CD3‐PerCp eFluor710 (eBioscience, clone SK7), Anti‐CD14‐A700 (BD, clone M5E2), Anti‐CD16‐PeCy7 (eBioscience, clone CB16), Anti‐HLADR‐APCH7 (BD, clone G46‐6), Anti‐CD11c‐APC (BD, B‐ly6).

### Statistical analysis

Comparisons between patient groups with HCV chronic evolution, spontaneous resolution, and exposed uninfected, during baseline, early acute, late acute, and follow‐up phases of infection, were evaluated by two‐way ANOVA (repeated measures). Data were analyzed with SigmaStat 3.5 for Windows (Systat Software, Inc., Chicago, IL). Comparisons between HCV chronic evolution, spontaneous resolution, exposed un‐infected, and healthy donors were evaluated by one‐way ANOVA. Non parametric tests were used if data did not pass normality test. Correlations were evaluated by Pearson's test if data passed normality test or by Spearman's test if data did not pass normality. Data were analyzed with GraphPad Prism 5.02 for Windows (GraphPad Software, San Diego, CA).

## Results

### Type III IFNs plasma levels and viral load during early acute HCV

**Figure 1:**
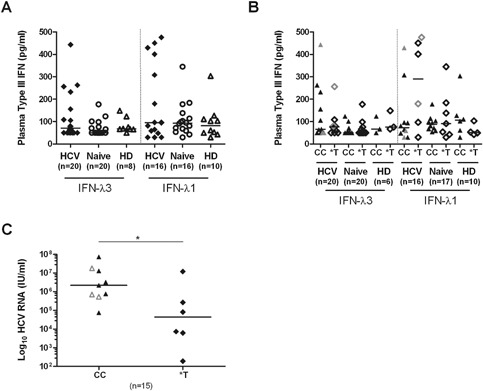
Type III IFNs plasma levels and viral load during early acute HCV. (A) Plasma levels of IFN‐λ3 and IFN‐λ1 were quantified by ELISA in HCV infected individuals during the early acute infection phase and in HCV naive PWID and HD. (B) Results of IFN‐λ3 and IFN‐λ1 plasma levels represented in (A) stratified by the favorable (CC) or the unfavorable (*T) IFN‐λ3 genotype, individuals with acute resolving infection are represented in gray symbols. (C) Viral load in HCV infected individuals during the early acute infection phase stratified by IFN‐λ3 genotype. Data under the limit of detection (≤100 IU/ml) are not represented; individuals with acute resolving infection are represented in gray open symbols. Median is represented by the horizontal bar. Statistical significance was tested by Mann–Whitney test *P < 0.05, **P < 0.01, ***P < 0.001.

To evaluate the role of type III IFNs during acute HCV, we compared plasma levels of IFN‐λ3 and IFN‐λ1 in a subset of HCV infected subjects during early acute infection (*n* = 20), in naïve PWID (*n* = 20) and in healthy donors (HD) (*n* = 8–10). Subjects were recruited and tested as described in Materials and Methods. Clinical characteristics and demographics are listed in Table 1. We observed high variability in type III IFNs plasma levels with a tendency towards higher levels in HCV acutely infected individuals but we did not observe significant differences among the groups tested (Fig. 1A) even when they were stratified according to their IFN‐λ3 favorable (CC) or unfavorable (*T) genotype (Fig. 1B). When we compared HCV viral loads in individuals with detectable RNA (>100 IU/ml) at the early acute time point, we observed significantly higher viral loads in individuals with CC as compared to the *T IFN‐λ3 genotype (*P* = 0.0256) (Fig. 1C) but there was no correlation between viral load and IFN‐λ3 levels (data not shown). These results suggest that early acute HCV is associated with variable type III IFNs plasma levels and that the favorable CC genotype is associated with higher viral load.

### IFN‐λ3 CC genotype is associated with decreased expression of NKG2A following spontaneous resolution

**Figure 2:**
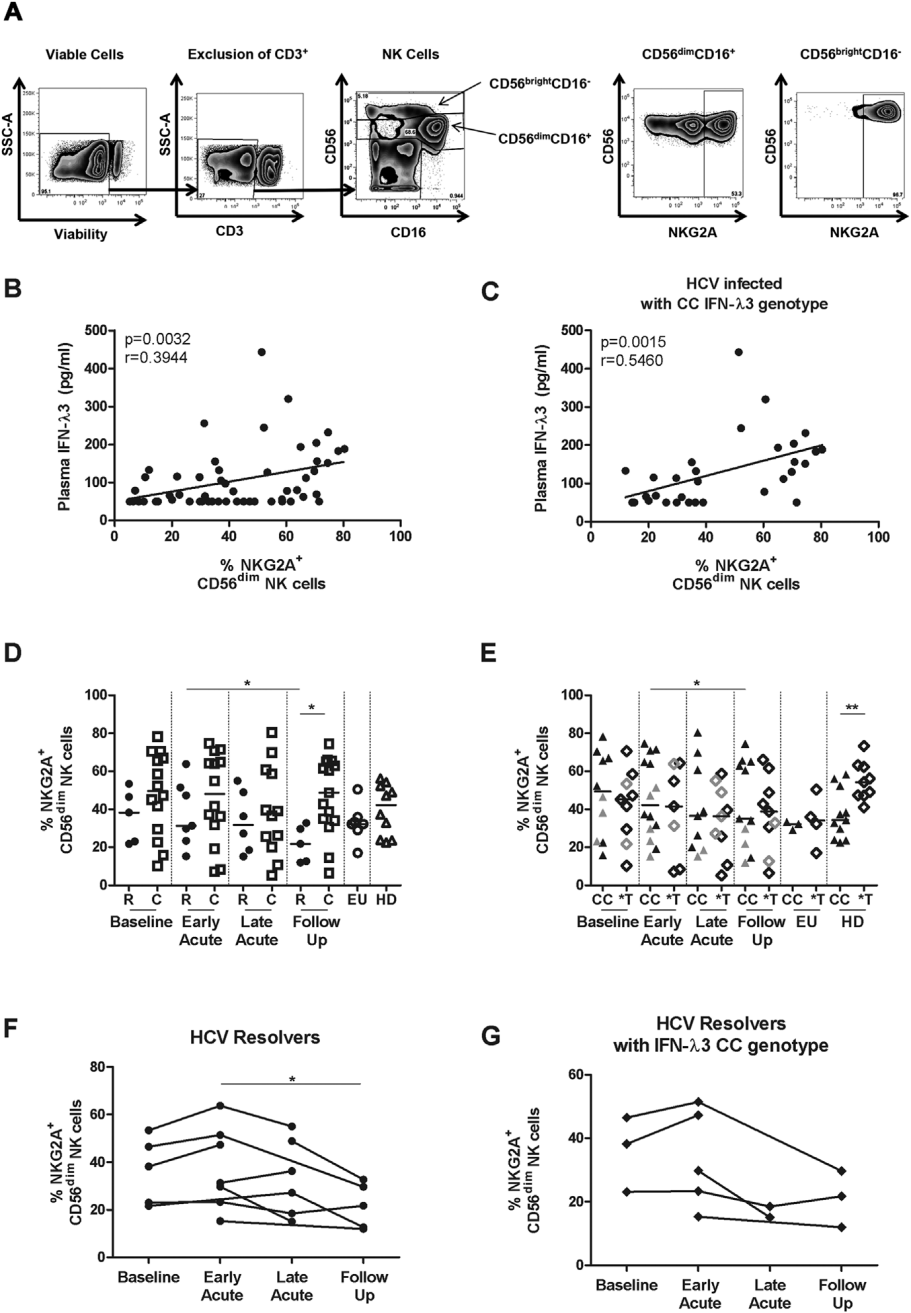
Reduced expression of NKG2A on CD56^dim^CD16+ NK cells correlates with lower IFN‐λ3 plasma levels and spontaneous resolution of acute HCV. (A) Gating strategy on the two NK cell subsets by flow cytometry: viable lymphocytes CD3‐CD56^dim^CD16+ and CD3‐CD56^bright^CD16‐. (B) IFN‐λ3 plasma levels correlates with expression of NKG2A on CD56^dim^CD16+ NK cells in all HCV infected individuals (Spearman Test) or (C) only those with the CC IFN‐λ3 genotype, where samples were available. (D) Frequency of NKG2A+ cells gated on CD56^dim^CD16+ NK cells is determined ex vivo in individuals with HCV chronic evolution (C), HCV spontaneous resolution (R), exposed uninfected (EU) and healthy donors (HD) at the indicated phases of infection, where samples were available. (E) Frequency of NKG2A+ cells gated on CD56^dim^CD16+ NK cells in HCV infected individuals, EU and HD, stratified according to the IFN‐λ3 genotype where samples were available. Individuals with HCV spontaneous resolution are represented in gray symbols. Early acute time point is represented for exposed uninfected individuals. Median is represented by a horizontal bar. (F) Longitudinal changes in the frequency of NKG2A+ cells gated on CD56^dim^CD16+ NK cells in individual patients in the spontaneous resolvers group. (G) Longitudinal changes in the frequency of NKG2A+ cells gated on CD56^dim^CD16+ NK cells in individual patients with the CC genotype in the spontaneous resolvers group. For each individual patient, different infection time points are joined by a line. Early acute phase is defined as 2 ± 1 month after the estimated time of infection and 1.5 month (range: 1–3 months) or after needle sharing for exposed uninfected; late acute phase is defined as 6 ± 2 months and Long‐term follow‐up is defined as 17 ± 6 months after the estimated time of infection. Two way ANOVA (repeated measures) or one‐way ANOVA (comparison with EU and HD). *P < 0.05, **P < 0.01, ***P < 0.001.

To determine whether the high variability observed in type III IFNs plasma levels during early acute HCV was associated with modulation of the phenotype of NK cells, we monitored longitudinal changes in expression of the NK cell receptors (KIR2DL1/S1 (CD158a), KIR2DL2/DL3 (CD158b), KIR3DL1 (NKB1), NKp30, NKp44, CD161 and NKG2D) on CD56^dim^CD16^+^, and CD56^bright^CD16^−^ NK cells. Flow cytometry gating strategy is represented in Figure 2A. There were no significant changes in expression of the majority of the receptors (Supplemental Fig. S1). We observed increased expression of NKG2D at the early acute and follow‐up time points as compared to baseline. However, due to limited sample availability at baseline when this marker was tested (*n* = 3), we considered this observation less relevant (Supplemental Fig. S1G). Nevertheless, when all patients were considered, increased NKG2D expression was observed in individuals carrying the CC genotype at the early acute and individuals carrying the *T genotype at both late acute and follow‐up time points (Supplemental Fig. S1H). There was no correlation between expression of any of the examined receptors and viral load or plasma levels of IFN‐λ3 except for NKG2A. The plasma levels of IFN‐λ3 at all the time points tested correlated positively with the percentage of CD56^dim^CD16^+^ cells expressing NKG2A (*P* = 0.0032, *r* = 0.3944) (Fig. 2B). However, there was no significant correlation when plasma levels of IFN‐λ3 at the early acute (*P* = 0.3528) or the late acute phase (*P* = 0.1215) were examined separately (data not shown). When individuals were stratified by their IFN‐λ3 genotype, the positive correlation between NKG2A expression and plasma IFN‐λ3 remained significant only in those with the CC genotype for CD56^dim^CD16^+^ (*P* = 0.0015, *r* = 0.5460) (Fig. 2C) and CD56^bright^CD16^−^ (*P* = 0.0378, *r* = 0.3748) (Supplemental Fig. S2A) subsets. The expression of NKG2A on both CD56^dim^CD16^+^ (*P* = 0.014) and CD56^bright^CD16^−^ (*P* = 0.009) NK cells decreased at the follow‐up time point following viral clearance in resolvers as we had previously described 17 (Fig. 2D and Supplemental Fig. S2B). Longitudinal data per patient from the resolver group is presented in Figure 2F demonstrates the full dynamics of NKG2A modulation before, during, and after acute HCV. Data for subjects with the CC genotype within the resolver group are represented in Figure 2G. When all acute HCV patients were stratified by IFN‐λ3 genotype, reduced NKG2A expression on the CD56^dim^CD16^+^ NK cells at the follow‐up time point remained significant only in individuals having the CC genotype (*P* = 0.028) (Fig. 2E). In HD, carriage of the CC IFN‐λ3 genotype was also associated with low NKG2A expression (*P* = 0.0035) suggesting a correlation between this genotype and lower NKG2A expression (Fig. 2E). These results indicate that decreased expression of NKG2A on NK cells, a marker of acute resolving HCV, was associated with lower IFN‐λ3 levels and the CC genotype.

### No correlation between IFN‐λ3 plasma levels and NK cells function during acute HCV

To evaluate whether IFN‐λ3 plasma levels and reduced NKG2A correlated with function of NK cells at different stages of infection, we monitored longitudinal changes in the cytotoxic potential of NK cells by analyzing the expression of CD107a, a degranulation marker and a surrogate marker of cytotoxicity 35, and IFN‐γ production in response to the NK cell target K562 cell line. There was no correlation between plasma levels of IFN‐λ3 (Supplemental Fig. S3) or IFN‐λ1 (data not shown) and the cytotoxic potential of both subsets of NK cells (Supplemental Fig. S3A and C) as well as their production of IFN‐γ cytokine (Supplemental Fig. S3B and D). These results suggest that IFN‐λ3 plasma levels do not directly correlate with function of NK cells.

### IFN‐λ3 CC genotype is associated with higher production of IFN‐γ by NK cells in chronics during early acute HCV

**Figure 3:**
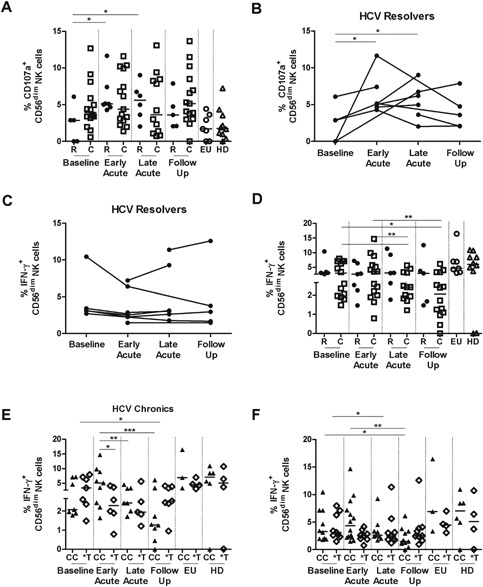
Degranulation and IFN‐γ production by CD56^dim^CD16^+^ NK cells during acute HCV. (A) Frequency of CD107a^+^IFN‐γ^−^ cells gated on CD56^dim^CD16^+^ NK cells was determined *ex vivo* in study subjects at the indicated phases of infection. (B, C) Longitudinal changes in the frequency of CD107a^+^IFN‐γ^−^ (B) or CD107a^−^IFN‐γ^+^ (C) cells gated on CD56^dim^CD16+ NK cells in individuals in the spontaneous resolvers group. For each individual patient, different infection time points are joined by a line. (D) Frequency of CD107a^−^IFN‐γ^+^ cells gated on CD56^dim^CD16^+^ NK cells was determined *ex vivo* in study subjects at the indicated phases of infection. (E) Frequency of CD107a^−^IFN‐γ^+^ cells gated on CD56^dim^CD16^+^ NK cells in acute HCV subjects with chronic evolution stratified according to the IFN‐λ3 genotype. (F) Frequency of CD107a^−^IFN‐γ^+^ cells gated on CD56^dim^CD16^+^ NK cells in acute HCV subjects stratified according to the IFN‐λ3 genotype. The first time point following exposure is represented for exposed uninfected individuals. Median is represented by the horizontal bar. Early acute, late acute and Long‐term follow‐up phases of infection are defined as in the legend to Figure 2. Two way ANOVA (repeated measures) or one‐way ANOVA (comparison with EU and HD). *P < 0.05, **P < 0.01, ***P < 0.001.

Next, we examined whether IFN‐λ3 genotype may still indirectly influence NK cell activity. As previously described during early acute HCV 17, we confirmed an increase in the capacity of CD56^dim^CD16^+^ to degranulate in individuals that resolved the infection (*P* = 0.011) (Fig. 3A). Longitudinal data per patient from the resolver group presented in Figure 3B demonstrates the full dynamics of CD107a expression before, during, and after acute HCV. This was not accompanied by an increase in the production of IFN‐γ but its stabilization in the majority of patients confirming the documented dissociation between these two functions 12, 36 (Fig. 3C and D). Progressive decrease in IFN‐γ production by CD56^dim^CD16^+^ NK subset during the progression from acute to the follow up phase was characteristic of infections progressing to chronicity (*P* = 0.002) (Fig. 3D). Similar results were observed for the CD56^bright^CD16^−^ subset upon progression from early to late acute infection (*P* = 0.033) (Supplemental Fig. S4A). When individuals with chronic evolution were stratified by IFN‐λ3 genotype, those with CC IFN‐λ3 genotype exhibited a trend similar to that observed in spontaneous resolvers with significantly higher production or rather stabilization of IFN‐γ by CD56^dim^CD16^+^ subset during the early acute phase (*P* = 0.040) (Fig. 3E). This effect was less evident when all patients were considered (Fig. 3F). This was not observed for the CD56^bright^CD16^−^ subset (Supplemental Fig. S4B). Finally, there was no correlation between viral load, serum ALT or the function of NK cells as was previously described 17 (data not shown). In summary, despite stabilization of IFN‐γ production by NK cells during early acute infection in individuals carrying the favorable CC genotype, suggesting an overall better NK cell function, this was not sufficient to control viral replication and many of these individuals developed persistent infection. This suggests that IFN‐λ3 genotype may influence NK cell function during acute HCV but other factors may contribute to determine the outcome of the infection.

### Effect of IFN‐λ3 stimulation on NK cells phenotype and function

**Figure 4:**
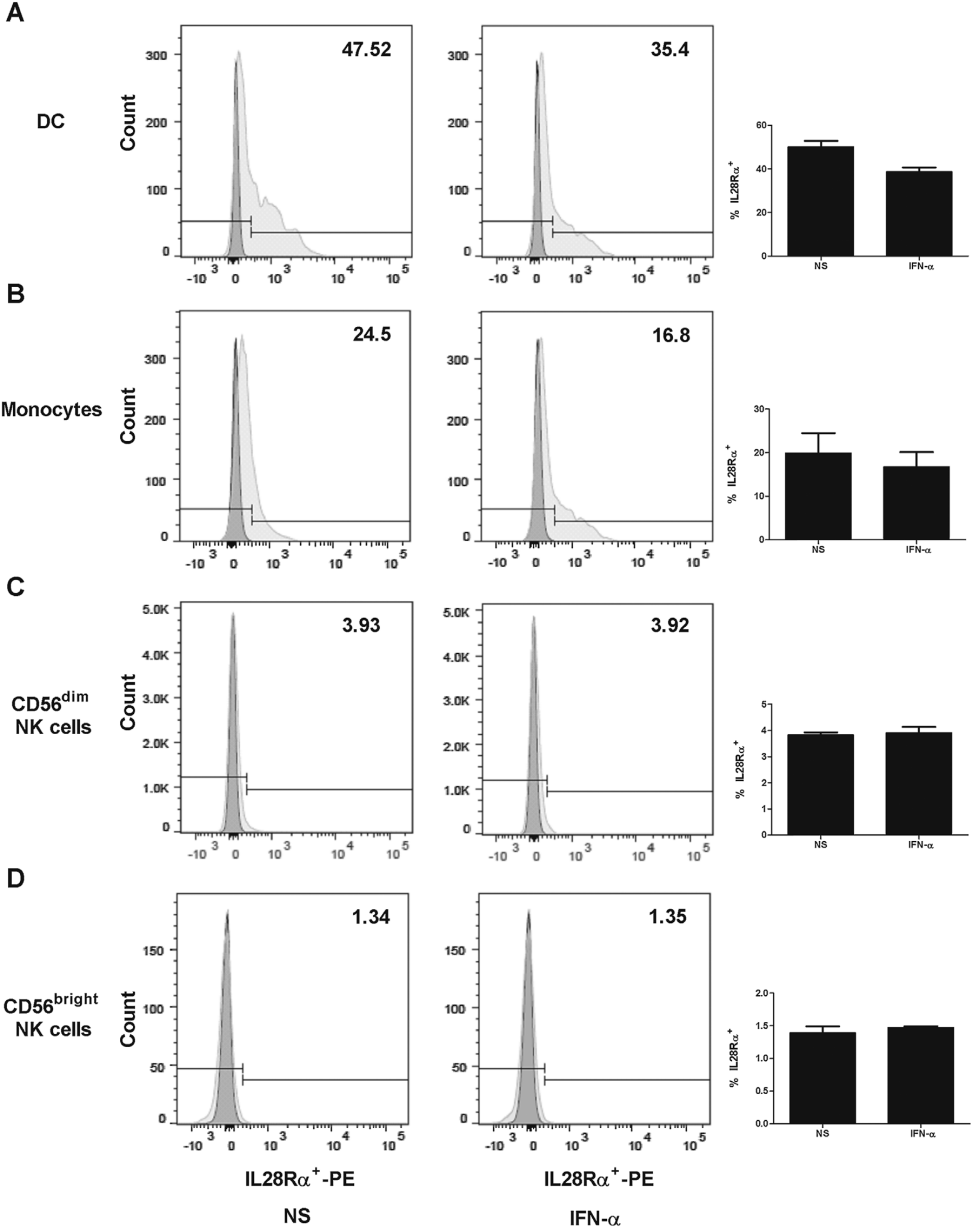
Stimulation with IFN‐α slightly affects the expression of IL28Rα on different lymphocyte subpopulations. PBMCs from 3 healthy donors were stimulated or not with IFN‐α and expression of IL28Rα was examined using flowcytometry as described in Materials and Methods. Left and middle panels show representative stainings on (A) dendritic cells (DCs) (CD3‐CD14‐CD16‐HLADR + CD11c+); (B) monocytes (CD3‐CD14 + CD16+); (C) CD56^dim^CD16+ NK cells and (D) CD56^bright^CD16‐. Fluorescence minus one (FMO) control staining for IL28Rα‐ PE is represented in dark grey and non‐stimulated (NS) and stimulated (IFN‐α) conditions for the different cell populations are represented in light grey in the representative histogram. Left panels represent the average expression on all three donors.

Given that we were not able to detect a direct correlation between IFN‐λ3 plasma levels, we hypothesized that it may act at the level of receptor regulation. IFN‐α stimulation of primary human hepatocytes (PHH) was reported to induce expression of the specific type III IFN receptor subunit (IL28Rα) at different levels depending on IFN‐λ3 genotype 37. However, studies reported controversial results about the expression of IL28Rα on different lymphocytes subsets 19, 23, 24, 25, 26, 27 and their subsequent ability to respond directly to IFN‐λ3 stimulation. To elucidate these controversies, we tested whether IFN‐α might directly affect the expression of IL28Rα on different lymphocytes subsets (Fig. 4). IL28Rα was highly expressed on DC (Fig. 4A) and monocytes (Fig. 4B and to a much lesser extent on CD56^dim^ NK cells (Fig. 4C) and CD56^bright^ NK cells (Fig. 4D). Stimulation of these different lymphocyte populations with IFN‐α did not significantly affect the expression of IL28Rα and may have even induced its slight down‐regulation.

We also tested whether IFN‐λ3 might directly affect expression of IL28Rα or NKG2A on NK cells or enhance their cytotoxic or cytokine producing functions. We stimulated whole PBMCs and purified NK cells from healthy donors (*n* = 10) with IFN‐λ3. We monitored changes in the expression of the surface receptor NKG2A and the specific subunit of the type III IFN receptor, IL28Rα by NK cells as well as the capacity of NK cells to degranulate in response to K562 cells and their IFN‐γ production before and after IFN‐λ3 stimulation. We did not observe any increase in the expression of IL28Rα (Supplemental Fig. S5A) or in NKG2A (Supplemental Fig. S5B) on CD56^dim^CD16^+^ cells following IFN‐λ3 stimulation. Also the stimulation did not affect the capacity of CD56^dim^CD16^+^ cells to degranulate (Supplemental Fig. S5C and data not shown) or to produce IFN‐γ (Supplemental Fig. S5D and data not shown). Similar data were observed for CD56^bright^CD16^−^ subset irrespective of the IFN‐λ3 genotype. These results suggest that IFN‐λ3 does not act directly on NK cells to influence their activation and function.

## Discussion

In this study we investigated the role of IFN‐λ3 polymorphism and plasma levels on activation and function of NK cells during acute HCV infection. Early acute HCV was characterized by high variability in type III IFNs plasma levels irrespective of IFN‐λ3 genotype, however, IFN‐λ3 CC genotype was predictive of higher viral load. Decreased expression of NKG2A on NK cells, a correlate of spontaneous HCV resolution, was associated with lower IFN‐λ3 plasma levels and the favorable CC genotype. IFN‐λ3 plasma levels did not directly correlate with degranulation and IFN‐γ production by NK cells. However, IFN‐λ3 genotype could still indirectly influence NK cell function where NK cells from patients with the CC genotype exhibited higher levels of IFN‐γ production but this was insufficient to prevent viral persistence. IFN‐λ3 stimulation by itself did not affect directly NK cell activation and function. These results suggest that IFN‐λ3 polymorphism indirectly affects NK cell phenotype and function during early acute HCV.

We observed high variability of IFN‐λ3 levels in the plasma during early acute HCV as was previously described in chimpanzees 6. However, we did not observe any statistically significant differences between patient groups for IFN‐λ1 plasma levels as in the chimpanzee model 6. It is possible that our results were skewed by the use of estimated date of the infection as compared to chimpanzee studies where timing of the infection was clearly documented. Our results are in agreement with the results of Langhans et al. 8 who did not observe significant differences in neither IL28B/IFN‐λ3 nor IL‐29/IFN‐λ1 in individuals with acute HCV as compared to healthy donors, although in that study acutely infected individuals with the CC genotype still exhibited higher levels of both cytokines as compared to TT and CT genotypes 8. We had previously reported that our cohort of PWIDs of a French Canadian origin had a higher representation of the CC and CT genotypes and reduced representation of the TT genotype as compared to other cohorts due probably to a strong genetic founder effect in that population 33. As such, we had very few subjects in the present study who were homozygous for the non‐favorable T allele (TT), so we had to group our TT subjects with the heterozygote CT subjects in one category as *T which may have influenced some of the results. Finally, induction of IFN‐λ3 mRNA in PBMCs following stimulation with Poly I:C was shown to correlate with polymorphisms in IFN‐λ4 rather than IFN‐λ3 genotypes 10. We genotyped all our participants for IFN‐λ4 (Table 1) and we observed no difference when results were analyzed and stratified according to the IFN‐λ4 favorable TTTT or non‐favorable *dG genotype. These results demonstrate that high variability of type III IFNs plasma levels is characteristic of the early acute phase of HCV infection irrespective of the IFN‐λ3 genotype.

Individuals with the CC IFN‐λ3 genotype exhibited higher viral load as compared to individuals with the *T IFN‐λ3 genotype during early acute infection as was previously described 38, 39, 40 but there was no correlation between viral load and plasma levels of IFN‐λ3 (data not shown). It was hypothesized that in individuals with the CC IFN‐λ3 genotype, viral replication might trigger a stronger innate and/or adaptive immune response that favor viral clearance 38. We examined HCV‐specific T cell responses in an IFN‐γ ELISPOT assay but observed no correlation between IFN‐λ3 plasma levels and the frequency of HCV‐specific T cells (data not shown) so we focused primarily on the role of the innate response represented by NK cells.

We observed a significant decrease in the expression of the NK cell inhibitory receptor NKG2A on NK cells following acute resolving HCV. This association was specific to individuals with the CC IFN‐λ3 genotype suggesting that CC IFN‐λ3 genotype might be predictive of reduced inhibition or better NK cell function. This may also explain why individuals with the CC IFN‐λ3 genotype have better prognosis following acute infection. Interestingly, it was previously reported that infected patients carrying the non‐favorable IFN‐λ3 TT allele expressed higher levels of NKG2A on effector NK cells before beginning peg‐IFN therapy and levels of expression correlated with reduced sustained viral response (SVR) rates 41. There was no correlation between plasma levels of IFN‐λ3 and NK cell function measured as the capacity to degranulate or to produce IFN‐γ. Similarly, stimulation of NK cells with IFN‐λ3 did not enhance NK cell degranulation of IFN‐γ production as was also recently reported 42. Nevertheless, carrying the favorable CC genotype was associated with higher levels of IFN‐γ production by NK cells during early acute infection in individuals who went chronic. Similar trends were observed by Rogalska‐Taranta et al. in chronic HCV patients undergoing IFN therapy 21. This suggests that although the favorable CC IFN‐λ3 genotype stabilizes IFN‐γ production by NK cells, this was not sufficient to mediate spontaneous clearance of HCV infection as these individuals became persistently infected.

It was reported that individuals co‐infected with HCV and human cytomegalovirus (HCMV) exhibited expansion of NKG2C+ NK cells concomitant with reduction in NKG2A+ cells 43. It is noteworthy that NKG2C was restricted to patients co‐infected with HCMV and not HCV monoinfected patients 43. It is also thought to distinguish a subset of HCMV reactive activated NK cells with memory characteristics 44. Further analysis of the concomitant expression of these two markers is required to understand their role in NK cell activation and function.

NK cell function during acute infection could also be dependent upon expression of KIR receptors (KIR2DL2 and KIR2DL3) on NK cells 16. KIR and IFN‐λ3 polymorphisms additively predict the response to HCV IFN‐based therapies 20 but given the limited number of patients in this study, we could not examine this correlation. Another key factor is the expression of the specific IL28Rα/ IFNλR1 on NK cells that has been a controversial issue. Overall, we observed low levels of receptor expression on NK cells and unlike hepatocytes 37 this expression was not modulated by stimulation with either IFN‐α or IFN‐λ3. Furthermore, IFN‐λ3 cytokine did not directly stimulate degranulation or IFN‐γ production by NK cells. Nevertheless, we cannot exclude that IFN‐λ3 may regulate NK cell differentiation or other functions. Indeed, IL28R was required for optimal NK cell activity in vivo 29. Additional assays are required to precisely assess the responsiveness of NK cells or other IL28Rα expressing cells to IFN‐λ3. Indeed, DCs and monocytes were shown to prime the activation of NK cells through direct interaction and/or production of different cytokines like IL‐12 45, 46. When we examined expression of IL28Rα on different lymphocyte populations, we observed higher expression of this receptor on DCs and monocytes as compared to NK cells prior to stimulation with IFN‐λ3 or IFN‐α, but there was no change in the level of expression following the stimulation. Recent reports suggest that IL‐12 and/or IL‐18 production by monocyte‐derived macrophages could act as the link between type III IFNs and activation of NK cells 26 and that this effect may be IFN‐λ3 genotype dependent 47. It will also be interesting to examine whether stimulation of NK cells using IL‐12/IL‐18 rather than K562 mediated activation would be directly influenced by IFN‐λ3 genotype.

The role of IFN‐λ4 during acute HCV remains to be elucidated. It might be induced during acute infection but its level of expression remained low 10. Degranulation of intrahepatic lymphocytes from HCV‐infected patients was associated with IFN‐λ4 polymorphisms 48. As discussed above, we observed no correlation between type III IFN plasma levels and IFN‐λ4 genotype (data not shown). When individuals were stratified according to their IFN‐λ4 genotype we observed the same trends for the modulation of NK cell phenotype or function as described for IFN‐λ3 genotypes (data not shown). Finally, IFN‐λ4 may not have a direct effect on NK cell activity since it binds to the same receptor as IFN‐λ3 which is expressed at a low level on NK cells 49.

In summary, our results suggest that IFN‐λ3 polymorphism may indirectly influence NK cell phenotype and function during acute HCV infection. Mechanisms underlying this indirect influence would require further investigation and may involve cross‐talk with DCs, monocytes, macrophages, and other lymphocyte populations or infected hepatocytes.

## Conflict of Interest

None declared.

## Supporting information

Additional supporting information may be found in the online version of this article at the publisher's web‐site

**Figure S1**. Longitudinal expression of NK cell marker and receptors on CD56^dim^CD16+ NK cells.
**Figure S2**. Decreased expression of NKG2A on CD56^bright^CD16‐ NK cells correlates with decreased IFN‐λ3 plasma levels in individuals with CC IFN‐λ3 genotype and spontaneous resolution of acute HCV.
**Figure S3**. IFN‐λ3 plasma levels do not correlate with NK cells activity during acute HCV.
**Figure S4**. Decreased production of IFNγ by CD56^bright^CD16‐ NK cells during acute HCV is not influenced by the IFN‐λ3 genotype.
**Figure S5**. Stimulation of NK cells with IFN‐λ3 does not affect CD56^dim^CD16+ NK cells activation and function.
