# Supplementary material for: IFN‐λ3 polymorphism indirectly influences NK cell phenotype and function during acute HCV infection
Source: Immun Inflamm Dis. 2016 Aug 16;4(3):376–88. doi: 10.1002/iid3.122 (PMC5004291; doi:10.1002/iid3.122)
Supplement: Supplementary file 1 — Figure S1. Longitudinal expression of NK cell marker and receptors on CD56dimCD16+ NK cells. Figure S2. Decreased expression of NKG2A on CD56brightCD16‐ NK cells correlates with decreased IFN‐λ3 plasma levels in individuals with CC IFN‐λ3 genotype and spontaneous resolution of acute HCV. Figure S3. IFN‐λ3 plasma levels do not correlate with NK cells activity during acute HCV. Figure S4. Decreased production of IFNγ by CD56brightCD16‐ NK cells during acute HCV is not influenced by the IFN‐λ3 genotype. Figure S5. Stimulation of NK cells with IFN‐λ3 does not affect CD56dimCD16+ NK cells activation and function. [file IID3-4-376-s001.docx]

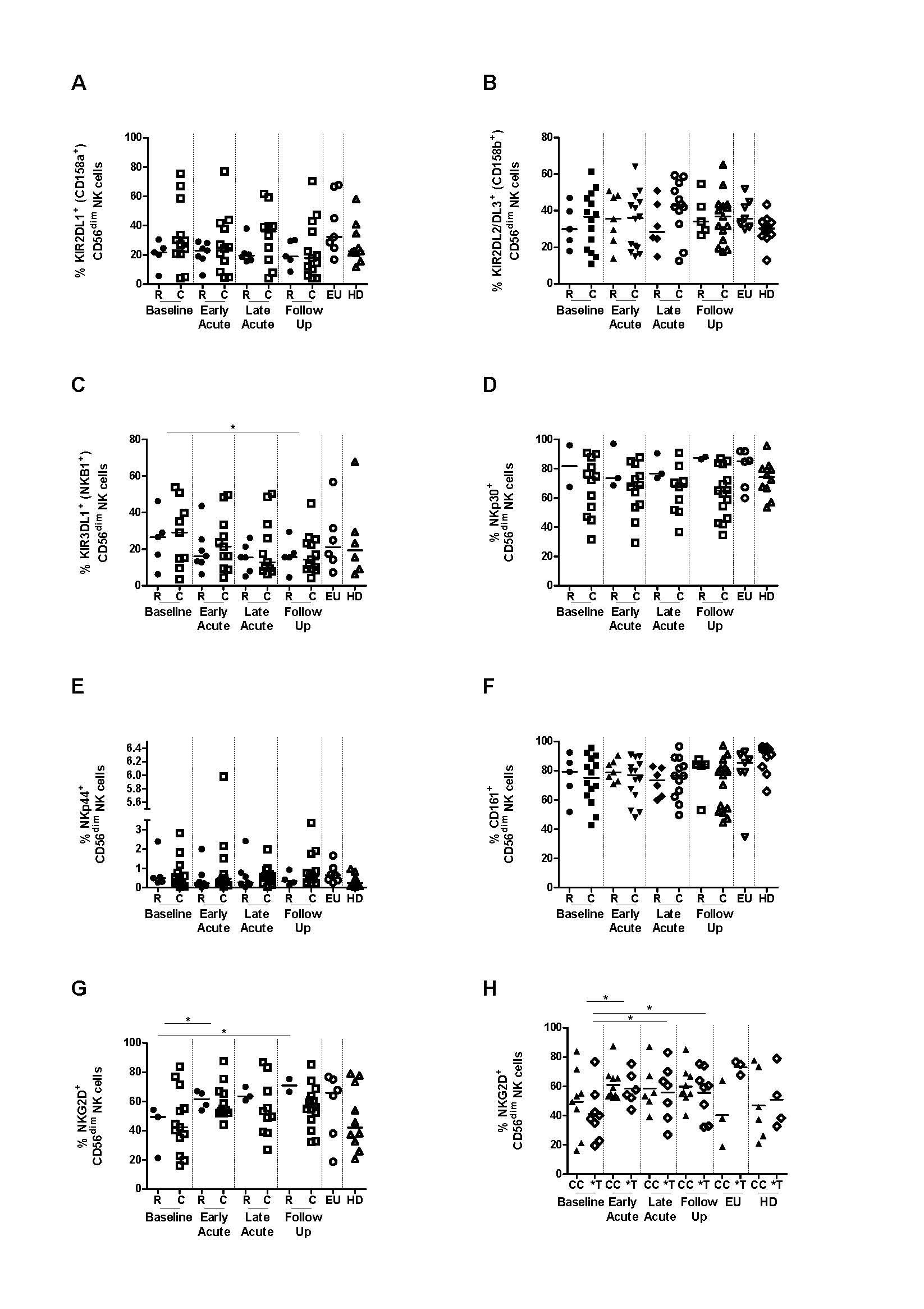


**Supplemental Figure S1: Longitudinal expression of NK cell marker and receptors on CD56^dim^CD16+ NK cells.** Frequency of (A) KIR2DL1 (CD158a), (B) KIR2DL2/DL3 (CD158b), (C) KIR3DL1 (NKB1), (D) NKp30, (E) NKp44, (F) CD161 and (G) NKG2D expressing CD56^dim^CD16+ NK cells was determined *ex vivo* in individuals with acute HCV progressing to either spontaneous resolution (R) or chronic infection (C), exposed uninfected (EU) and healthy donors (HD) at the indicated phases of infection where samples were available. (H) Frequency of NKG2A+ cells gated on CD56^dim^CD16+ NK cells in HCV infected individuals, EU and HD, stratified according to the IFN-λ3 genotype where samples were available. Early acute time point is represented for exposed uninfected individuals. Median is represented by a horizontal bar. Early acute, late acute and long-term follow-up phases of infection are defined as described in Figure2. Two way ANOVA (repeated measures) or one-way ANOVA (comparison with EU and HD) were used. *p<0.05, **p<0.01, ***p<0.001.


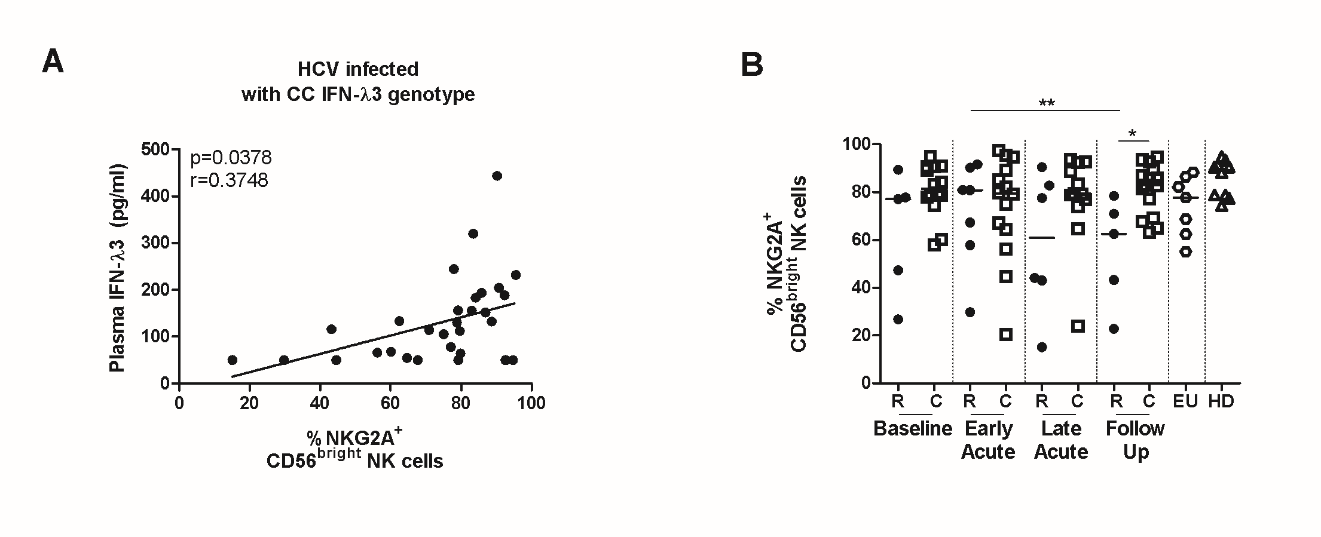


**Supplemental Figure S2: Decreased expression of NKG2A on CD56^bright^CD16- NK cells correlates with decreased IFN-λ3 plasma levels in individuals with CC IFN-λ3 genotype and spontaneous resolution of acute HCV.**

(A) IFN-λ3 plasma levels at all the time points studied correlates with expression of NKG2A on CD56^bright^CD16- NK cells in HCV infected individuals with the favorable (CC) IFN-λ3 genotype (Spearman Test). (B) Frequency of NKG2A+ cells gated on CD56^bright^CD16- NK cells measured *ex vivo* in study subjects at the indicated phases of infection. The first time point following exposure is represented for exposed uninfected individuals. Median is represented by the horizontal bar. Two way ANOVA (repeated measures) or one-way ANOVA (comparison with EU and HD). *p<0.05, **p<0.01, ***p<0.001.


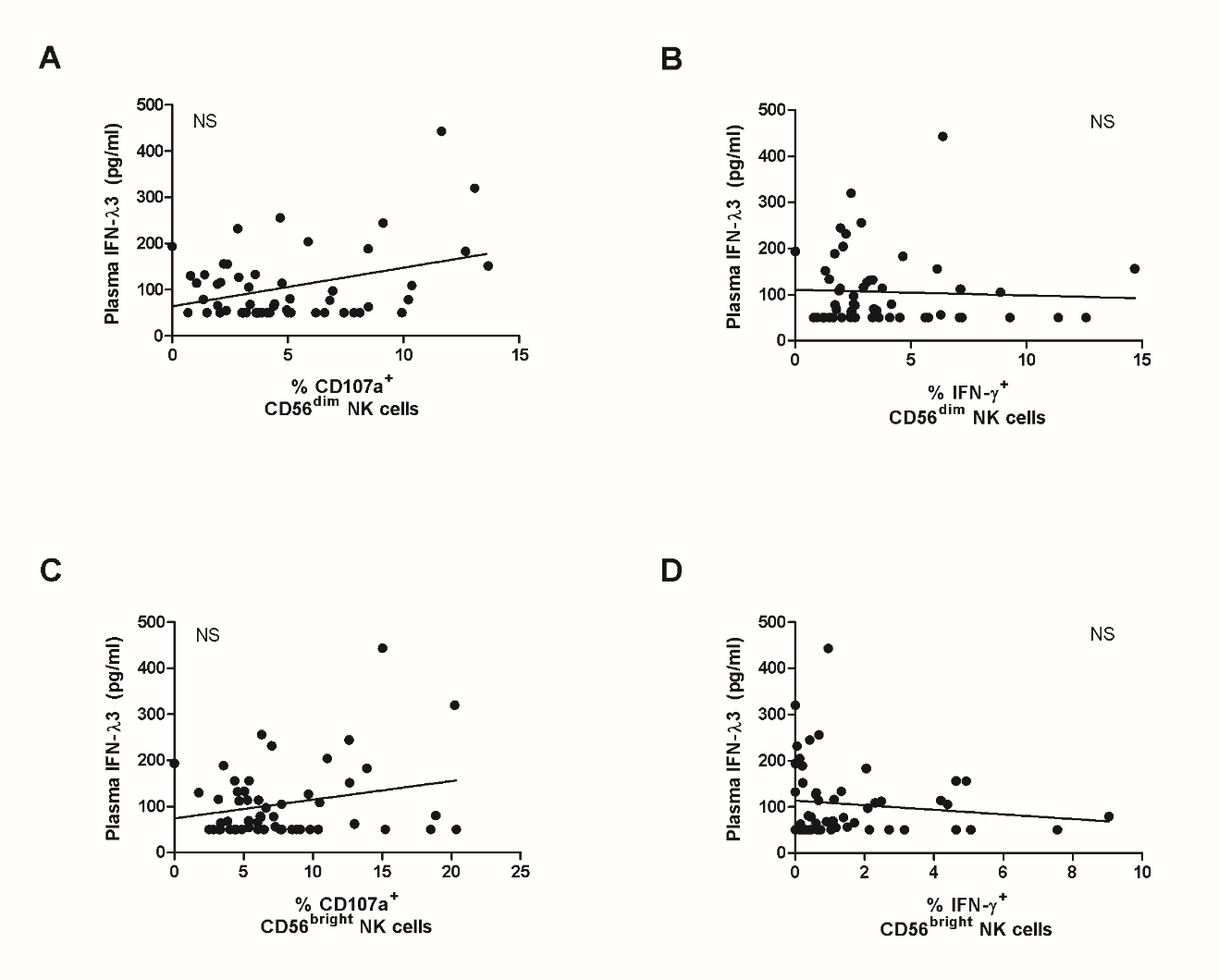


**Supplemental Figure S3: IFN-λ3 plasma levels do not correlate with NK cells activity during acute HCV.**

IFN-λ3 plasma levels in HCV infected individuals do not correlate with (A,C) expression of CD107a or (B,D) production of IFN-γ by CD56^dim^CD16^+^ (A,B) or CD56^bright^CD16^-^ (C,D) NK cells upon stimulation with K562 cells at all the time points tested (Spearman Test). (NS: Not significant).


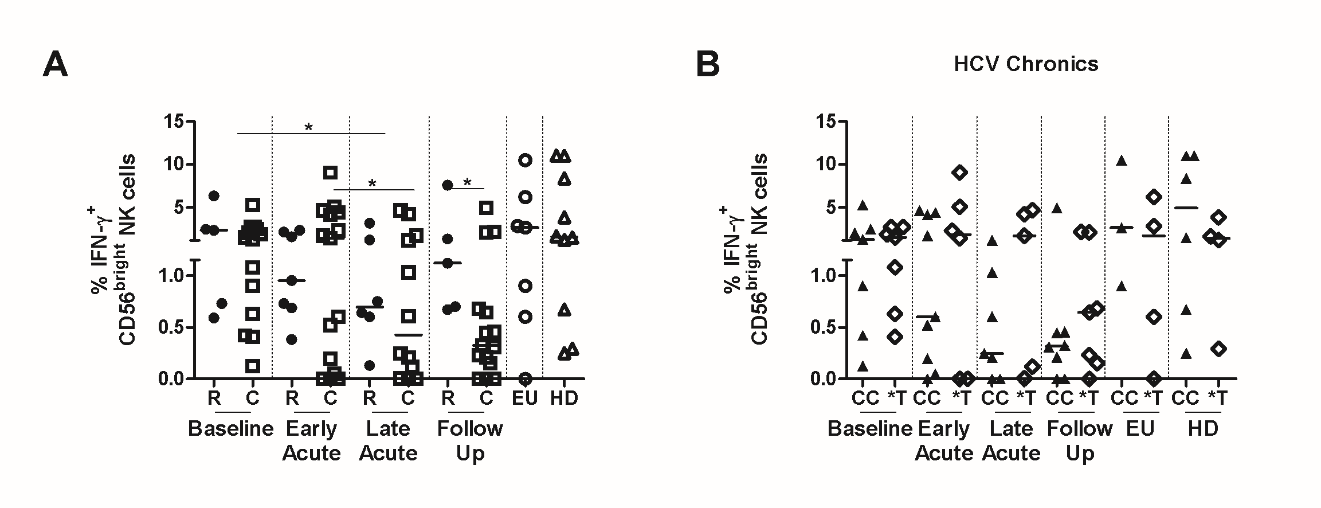


**Supplemental Figure S4: Decreased production of IFNγ by CD56^bright^CD16^-^ NK cells during acute HCV is not influenced by the IFN-λ3 genotype.**

(A) Frequency of CD107a^-^IFN-γ^+^ cells gated on CD56^bright^CD16^-^ NK cells is determined *ex vivo* in individuals with HCV chronic evolution (C), HCV spontaneous resolution (R), exposed uninfected (EU) and healthy donors (HD) at the indicated phases of infection. (B) Frequency of CD107a^-^IFN-γ^+^ cells gated on CD56^bright^CD16^-^ NK cells in individuals with HCV chronic evolution stratified according to the IFN-λ3 genotype. Early acute time point is represented for exposed uninfected individuals. Median is represented by a horizontal bar. Two way ANOVA (repeated measures) or one-way ANOVA (comparison with EU and HD). *p<0.05, **p<0.01, ***p<0.001.


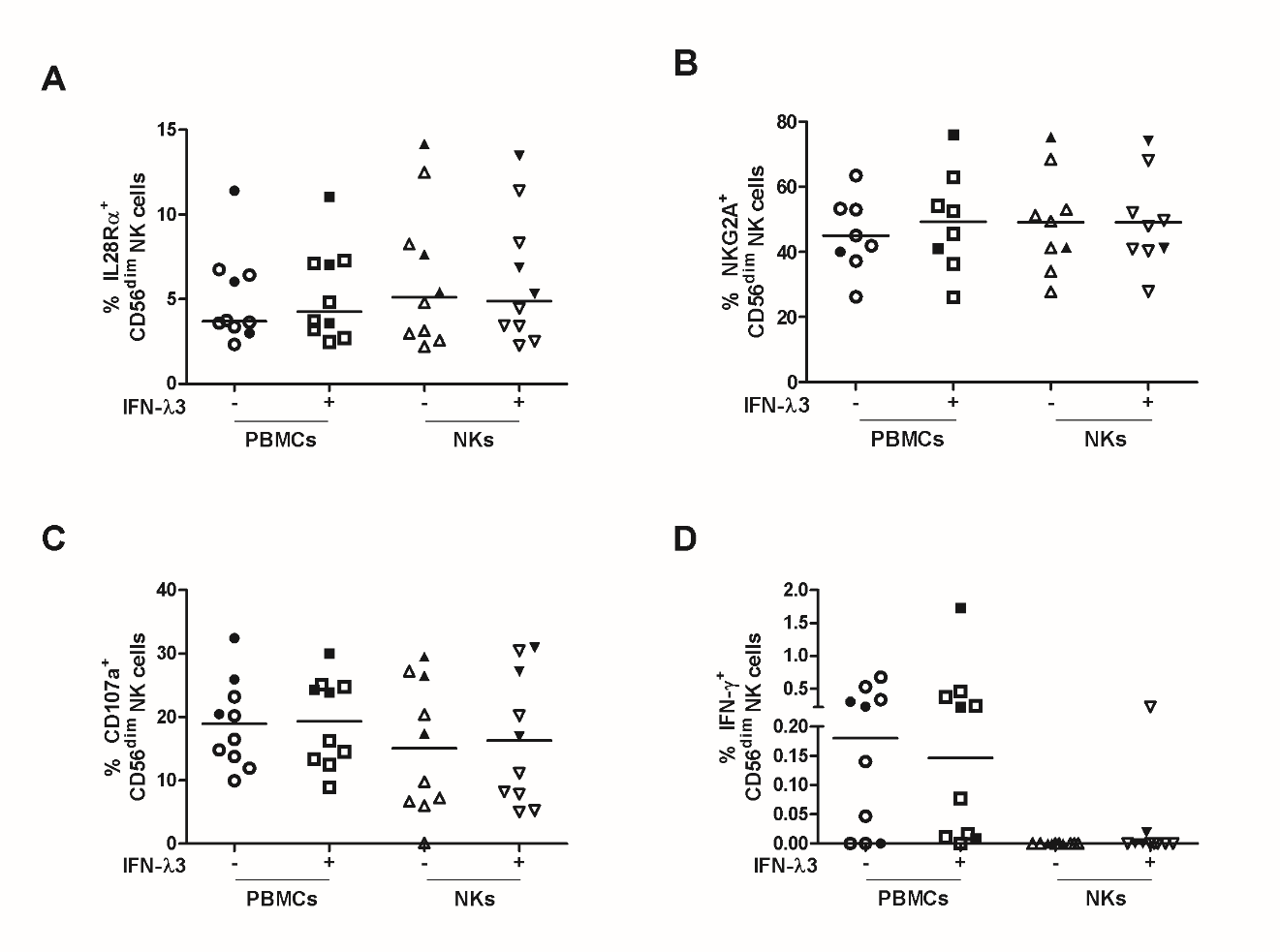


**Supplemental Figure S5. Stimulation of NK cells with IFN-λ3 does not affect CD56^dim^CD16^+^ NK cells activation and function.**

(A) Frequency of IL28Rα^+^ cells and (B) Frequency of NKG2A^+^ cells gated on CD56^dim^CD16^+^ NK cells measured *ex vivo* in PBMCs or purified NK cells from healthy donors stimulated or not with IFN-λ3. (C) Frequency of CD107a^+^ cells or (D) Frequency of IFN-γ^+^ cells within CD56^dim^CD16^+^ NK cells in presence of K562 as measured in PBMCs or purified NK cells from healthy donors stimulated or not with IFN-λ3. Individuals with CC IFN-λ3 genotype are represented in open symbols. Median is represented by the horizontal bar. Statistical significance was tested by paired t-test *p<0.05, **p<0.01, ***p<0.001.
